# Gerrymandering and computational redistricting

**DOI:** 10.1007/s42001-019-00053-9

**Published:** 2019-08-13

**Authors:** Olivia Guest, Frank J. Kanayet, Bradley C. Love

**Affiliations:** 1 Department of Experimental Psychology, University College London, London, UK; 2 Department of Psychology, Stanford, USA; 3 The Alan Turing Institute, London, UK

**Keywords:** Gerrymandering, Computational redistricting, Weighted *k*-means, Cognitive limitations

## Abstract

Partisan gerrymandering poses a threat to democracy. Moreover, the complexity of the districting task may exceed human capacities. One potential solution is using computational models to automate the districting process by optimizing objective and open criteria, such as how spatially compact districts are. We formulated one such model that minimised pairwise distance between voters within a district. Using US Census Bureau data, we confirmed our prediction that the difference in compactness between the computed and actual districts would be greatest for states that are large and, therefore, difficult for humans to properly district given their limited capacities. The computed solutions highlighted differences in how humans and machines solve this task with machine solutions more fully optimised and displaying emergent properties not evident in human solutions. These results suggest a division of labour in which humans debate and formulate districting criteria whereas machines optimise the criteria to draw the district boundaries. We discuss how criteria can be expanded beyond notions of compactness to include other factors, such as respecting municipal boundaries, historic communities, and relevant legislation.

## Introduction

One of the greatest threats to democracy, particularly in the USA, is gerrymandering. Gerrymandering is the practice of (re)drawing electoral district boundaries to advance the interests of the controlling political faction. The term is a portmanteau, coined in 1812 when people noticed that a district—approved by the then governor of Massachusetts, Elbridge Gerry—resembled a salamander [20].

Gerrymandering leads to districts with unnecessarily visually complex shapes, e.g. North Carolina (see Fig. 3c). Although there are laws (both at the state and federal levels) to safeguard the rights of citizens (including minorities) during the redistricting process, in practice these laws do little to reduce partisan gerrymandering [15]. Worryingly, gerrymandering is on the rise [25] due to partisan actions of both Republicans and Democrats [5]. In the 17 states where Republicans controlled the redistricting process, they secured 72% of the available seats on only 52% of the vote. Mirroring, in the six states where Democrats controlled the districting process, they secured 71% of the seats on 56% of the vote.

**Fig. 1:**
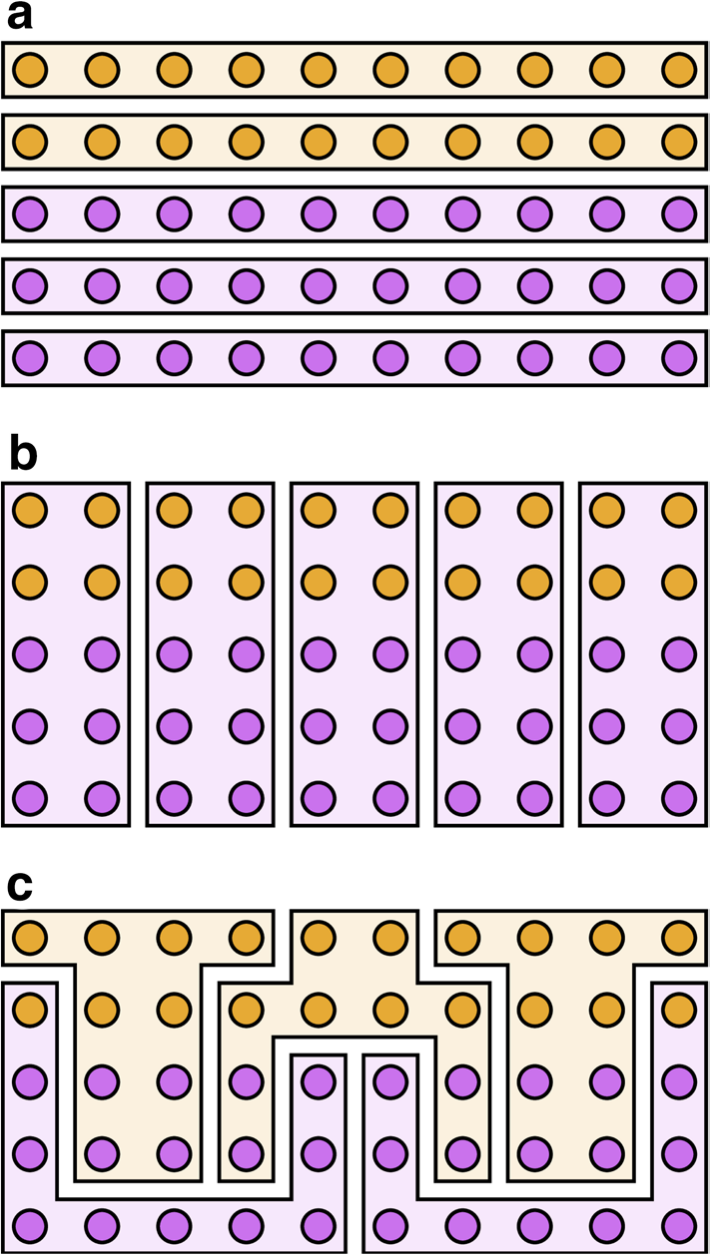
An illustrative example of three redistricting plans. The 50 voters (circles) are grouped into 5 districts (polygons) with the background colour denoting the winning party. The purple party (60% of voters) secures 60%, 100%, and 40% of the seats under the three plans, respectively. **a** Compact, fair: the proportion of wins (60%) by the purple party reflects its overall level of voter support. **b** Compact, not fair: all five districts are won by the purple party because the orange vote has been cracked. **c** Not compact, not fair: the purple party has been packed into two districts (its only wins) and cracked in the remaining districts. We recommend the video that motivated this figure [26]

The two main gerrymandering strategies are packing and cracking [1]. Cracking dilutes people likely to vote for the opposition, assigning them to as many districts as possible, see Fig. 1b. One cracking tactic is to dilute urban voting blocs by having multiple districts from the countryside converge like the spokes of a wheel at a city’s fractured hub. In contrast, packing concentrates people who will likely vote for the opposition within a small number of districts, rendering their vote inconsequential in the remaining districts, see Fig. 1c.

One possible solution to partisan gerrymandering is to rely on computer algorithms to impartially draw districts [27]. In theory, there is no reason why such a solution could not be adopted in the USA. Indeed, many states within Mexico use computer algorithms to district [2, 11, 13, 24]. Moving to computational redistricting would “elevate the legislative redistricting debate from a battle over line drawing to a discussion of representational goals” [6, p. 1381]. In other words, the role for humans would be to decide and formalise the criteria (e.g. people within a district should be close to one another) and the computer’s job would be to find the best solution without human tinkering. Thus, the appeal of automation is twofold: (a) open source computer algorithms can be written to follow objective criteria absent corrupting influences; (b) computers are able to toil away optimising the objective criteria in contrast to humans who have limited cognitive capacity and time to devote. While the first point, namely that purposeful gerrymandering occurs and leads to unfair solutions may be obvious, the second point may be less so. However, from a psychological perspective, it is clear that humans do not consider all logically possible solutions in combinatoric problems (e.g. districting), but may instead rely on shortcuts and general organisational principles [23]. Counterintuitively, some of what we perceive as gerrymandering may simply reflect that humans are not very good at the districting task.

In light of these observations, we tested the psychologically motivated prediction that differences in compactness between the computed and actual districts will be greatest for states that are large and, therefore, difficult for humans to properly district. To evaluate this prediction, we devised a novel clustering algorithm to redistrict the USA’s 435 congressional districts (more populous states are allotted more seats). The algorithm maximises a notion of compactness by minimising the average mean distance between people within the same district, cf. [3, 8, 14].

In accord with federal law, our novel algorithm includes an additional constraint to create clusters (i.e. districts) of roughly the same cardinality (i.e. population). We refer to our algorithm as weighted *k*-means because it is based on *k*-means clustering [4, 18]. Details are provided in Materials and methods and the open source code is available to reproduce the reported results at https://osf.io/5fepu/.

## Materials and methods

This section details how the US Census Bureau data were preprocessed and provides details on the weighted *k*-means model.

### Census data

US Census Bureau data were used to perform the district clusterings reported in the main text. For clustering, we used the smallest available geographic unit, known as a census block. The US Census Bureau collects data for just over 11 million census blocks of which almost 5 million have a population of 0. The last decennial census occurred in 2010. However, as recently as 2015, the US Census Bureau conducted the ACS (American Community Survey), which is a survey at one level above the block level, which is referred to as a block group. Using these 2015 counts, we estimated the population of each census block in 2015 by calculating its population share of its block group in 2010 and, assuming these proportions had not changed, updated the block populations based on the 2015 ACS. Notice that our population estimates for census blocks in 2015 is not constrained to be an integer.

Census blocks in urban areas tend to be geographically smaller but more populated. Based on our estimates combining the 2010 and 2015 data, the mean population of a census block is 29.49 with a median of 3.41 people. The mean area of a census block 1.11 $\hbox {km}^2$ with a median of 0.04 $\hbox {km}^2$.

### Initialisation

The manner in which clusters are initialised will affect the quality of the final solution because our algorithm, like *k*-means which it generalises, moves toward a local optimum. We initialise the centroids using the procedure from *k*-means++ [4].

### Weighted *k*-means algorithm

Weighted *k*-means generalises *k*-means by preferring clustering solutions in which clusters have roughly the same cardinality (i.e. number of members) with the strength of this preference determined by a parameter value. This scaling factor is necessary to ensure that clusters (i.e. districts) have roughly the same number of voters, which is fair and required by federal law. In effect, the scaling makes it more likely that clusters with fewer members will geographically expand to encompass more members (see parameter fitting for how we found values for the scaling factor).

Like *k*-means, in each iteration, items are assigned to the nearest cluster and at the end of iteration the position of the cluster (i.e. centroid) is updated to reflect its members’ positions. After a number of iterations, the algorithm converges to a local optimum. Weighted *k*-means differs from *k*-means by penalising clusters with more members such that distances to these clusters are multiplied by a scaling factor reflecting the cluster’s cardinality. The weight for cluster *i* is

1 $$\begin{aligned} w_i = \frac{|C_i|^{\alpha }}{\sum \nolimits _{j=1}^{K}{|C_j|^{\alpha }}} , \end{aligned}$$

where $|C_i|$ is the cardinality of cluster *i*, *K* is the number of clusters, and $\alpha$ is a parameter that determines how much to penalise clusters with a disproportionate number of members.

To stabilise solutions across iterations and prevent oscillations, the scaling factor ${s}_{i, t}$ for cluster *i* at time *t* (i.e. iteration *t*) is calculated as a weighted combination of the previous scaling factor ${s}_{i, t-1}$ and $w_i$

2 $$\begin{aligned} {s}_{i, t}= \beta {s}_{i, t-1} + (1-\beta ) w_i , \end{aligned}$$

where $\beta$ is a control parameter in the range [0, 1). In the first iteration, each ${s}_{i, 0}$ is initialised to $\frac{1}{K}$, where *K* is the number of clusters.

The scaled distance of point *x* to the cluster *i* is

3 $$\begin{aligned} d_{s}(x, \mu _i) = s_{i,t} \times d(x, \mu _i) , \end{aligned}$$

where $\mu _i$ is the position of cluster *i* and *d* is the distance metric, which in this contribution is great-circle distance (also known as orthodromic or geodesic distance, estimated using the haversine formula), which respects the curvature of the Earth. 
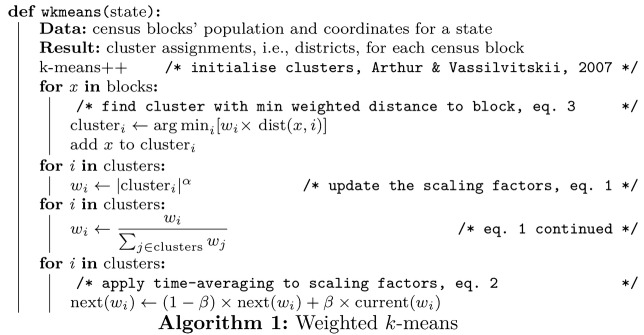
 Finally, $\text {argmin}_i d_{\text {s}}(x, \mu _i)$ is used to find the nearest weighted cluster *i* from point *x*, to which *x* will be assigned. Notice that this algorithm is identical to *k*-means when $\alpha$ is 0. As $\alpha$ increases, the constraint of equal cardinality becomes firmer. The algorithm is presented in pseudocode in Algorithm 1.

### Parameter fitting

Solutions are only considered that converge and for which the cardinalities of the clusters are in line with that of actual congressional districts. In principle, one could use any parameter search procedure to find $\alpha$ and $\beta$ that minimised the measure we report, which is the pairwise distance of voters within a district (i.e. cluster). For example, one could use grid search to consider all possible combinations (at some granularity) of $\alpha$ and $\beta$.

However, given available computing resources, we adopted a more efficient procedure informed by our understanding of the algorithm’s behaviour (i.e. smaller $\alpha$ values lead to tighter clusterings). The parameter search procedure began with $\alpha$ set to 0 and increased $\alpha$ until an acceptable solution was found. At each level of $\alpha$, $\beta$ was set to 0.5 and increased by 0.1 after a simulation failure until $\beta$ exceeded its range. At that point, $\alpha$ was increased by 0.1 and the process was repeated with $\beta$ set to 0.5. This procedure terminated when an acceptable solution was found. At that point, a finer grained optimisation was performed, which considered $\alpha$ values up to 0.1 lower than first acceptable value found.

## Results

**Fig. 2:**
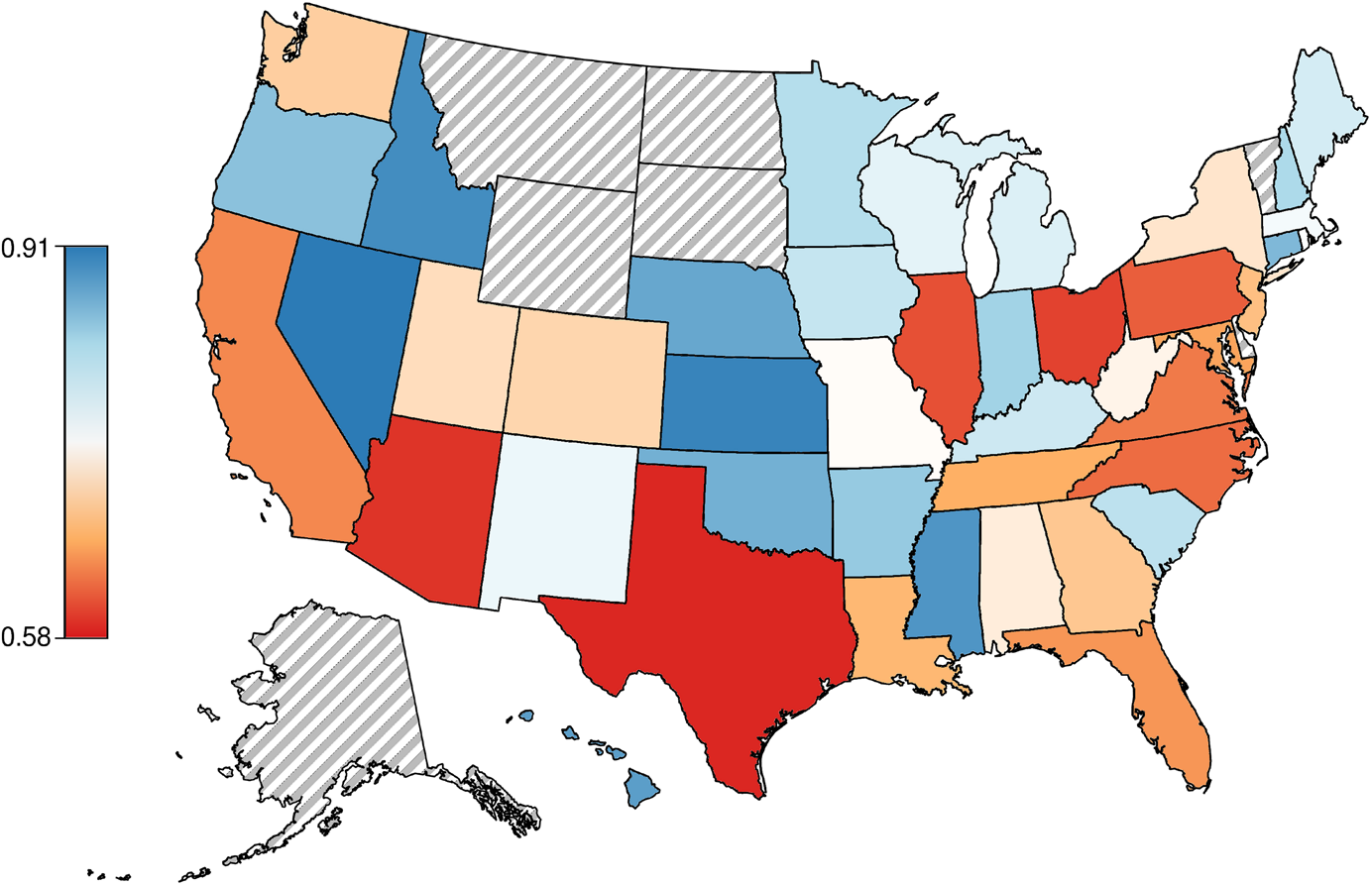
Map of the USA showing how much more compact each state’s districts would be under computational districting. Red-coloured states would improve the most after using our algorithm to form districts that are compact by minimising the pairwise distances between people within a district. Blue-coloured states would improve the least from computational redistricting, though still show an improvement in within-district pairwise distances. States with grey hatching, e.g. Alaska (bottom left), have only one district

Our clustering algorithm created improved maps for every state, see Fig. 2. Please visit http://redistrict.science to compare the actual and automated districting plans for any address in the USA. We define the improvement for each state as the ratio of pairwise distances within districts between our solution and the actual districts. This metric favours districts in which voters are tightly clustered spatially, leading to a mean improvement across states of 0.796 (i.e,. about 20%) with standard deviation 0.0858. To test our main prediction, a regression model was fit to the state improvement scores with number of districts, and square of number of districts serving as predictors, $R^2 = 0.550$, $F(2, 40) = 24.47,\, p \approx 0$. Both predictors in the fit, $-\, 0.0149(\text {number of districts})\, +\, 0.0002(\text {number of districts})^2 +\, 0.9027$, were statistically significant, $t(40) = -5.654,\, p \approx 0.0$ and $t(40) = 3.879,\, p \approx 0$, respectively. Consistent with our prediction, these results suggest that the cognitive demands of drawing districts for larger states may tax human capacities. Thus, some of the unfairness in current solutions may be unintentional, as opposed to wholly attributable to deliberate gerrymandering for political gain.

The regression also included a quadratic term for the number of districts that confirmed the intuition that the complexity of the task should not scale linearly with the number of districts because the clustering is spatial and local interactions dominate. For instance, there are natural groupings and locality within big states, e.g. what is drawn for Los Angeles is unlikely to strongly affect what is drawn in San Francisco. People likely segment maps hierarchically into regional groupings to reduce processing demands, as they do in other map reasoning tasks [12], which may explain fallacious conclusions like that Reno lies east of Los Angeles and that Atlanta is east of Detroit [22]. Overall, the district size results indicate that states with fewer districts are easier to draw properly, which suggests that state size may be another cause of “accidental gerrymandering” [8]. The residuals from this regression model can be interpreted as how gerrymandered each state is, adjusting for population. This analysis suggests that Arizona is the most gerrymandered state (see Table 1 for the complete ranking).

**Table 1:** States sorted by their residuals from the regression model described in the main text

State	Residuals	State	Residuals	State	Residuals
AZ	− 0.1482	ME	− 0.0329	NE	0.0267
MD	− 0.0820	NM	− 0.0220	OR	0.0399
LA	− 0.0792	NH	− 0.0158	SC	0.0401
OH	− 0.0747	WA	− 0.0122	WI	0.0419
VA	− 0.0747	NJ	− 0.0043	CT	0.0426
UT	− 0.0632	CA	− 0.0036	MA	0.0437
TX	− 0.0623	IA	− 0.0022	MS	0.0458
NC	− 0.0551	AL	0.0027	OK	0.0527
IL	− 0.0538	HI	0.0137	MN	0.0563
TN	− 0.0503	GA	0.0181	FL	0.0568
PA	− 0.0475	KY	0.0203	KS	0.0603
WV	− 0.0466	ID	0.0217	NV	0.0628
RI	− 0.0458	MO	0.0239	IN	0.0813
CO	− 0.0386	AR	0.0244	MI	0.1047
				NY	0.1345

A state’s residual can be interpreted as how gerrymandered the state is after taking into account the number of districts, with negative residuals indicating greater gerrymandering. Of course, there could be other important covariates in addition to population size

**Fig. 3:**
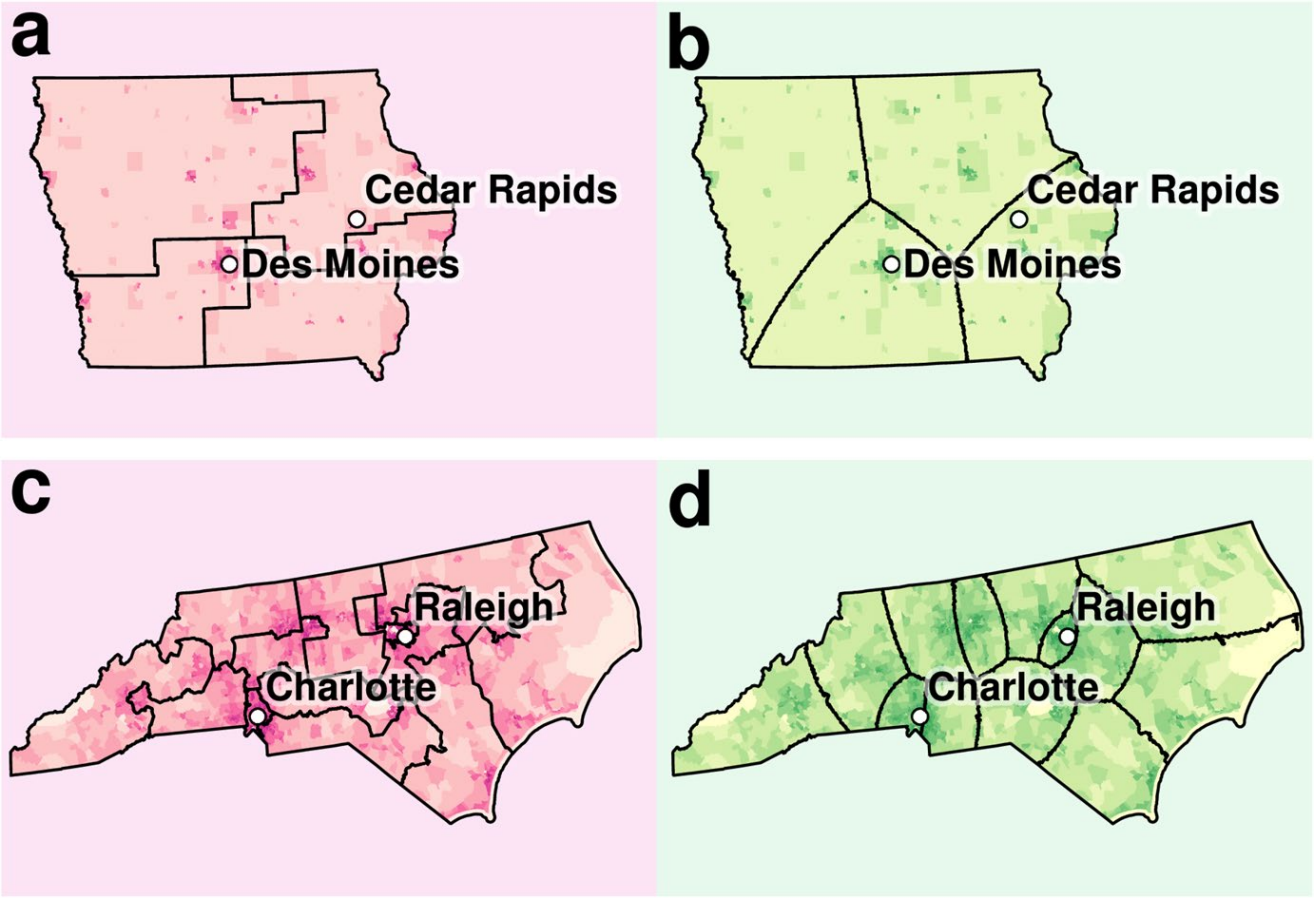
Actual and computed district maps for Iowa (**a**, **b**) and North Carolina (**c**, **d**). Computed solutions are shown in green to the right of the actual congressional districts. Darker areas on the map (census tracts) are more densely populated

Let us turn to some specific examples for redistricting solutions (for an interactive map, visit http://redistrict.science). For Iowa, which uses a neutral commission to draw district boundaries [16], our automated solution uses fewer segments (Fig. 3b) than the more complex actual solution (Fig. 3a). In the case of North Carolina, where maps are drawn through a partisan process, improvements are also evident (Fig. 3c, d).

**Fig. 4:**
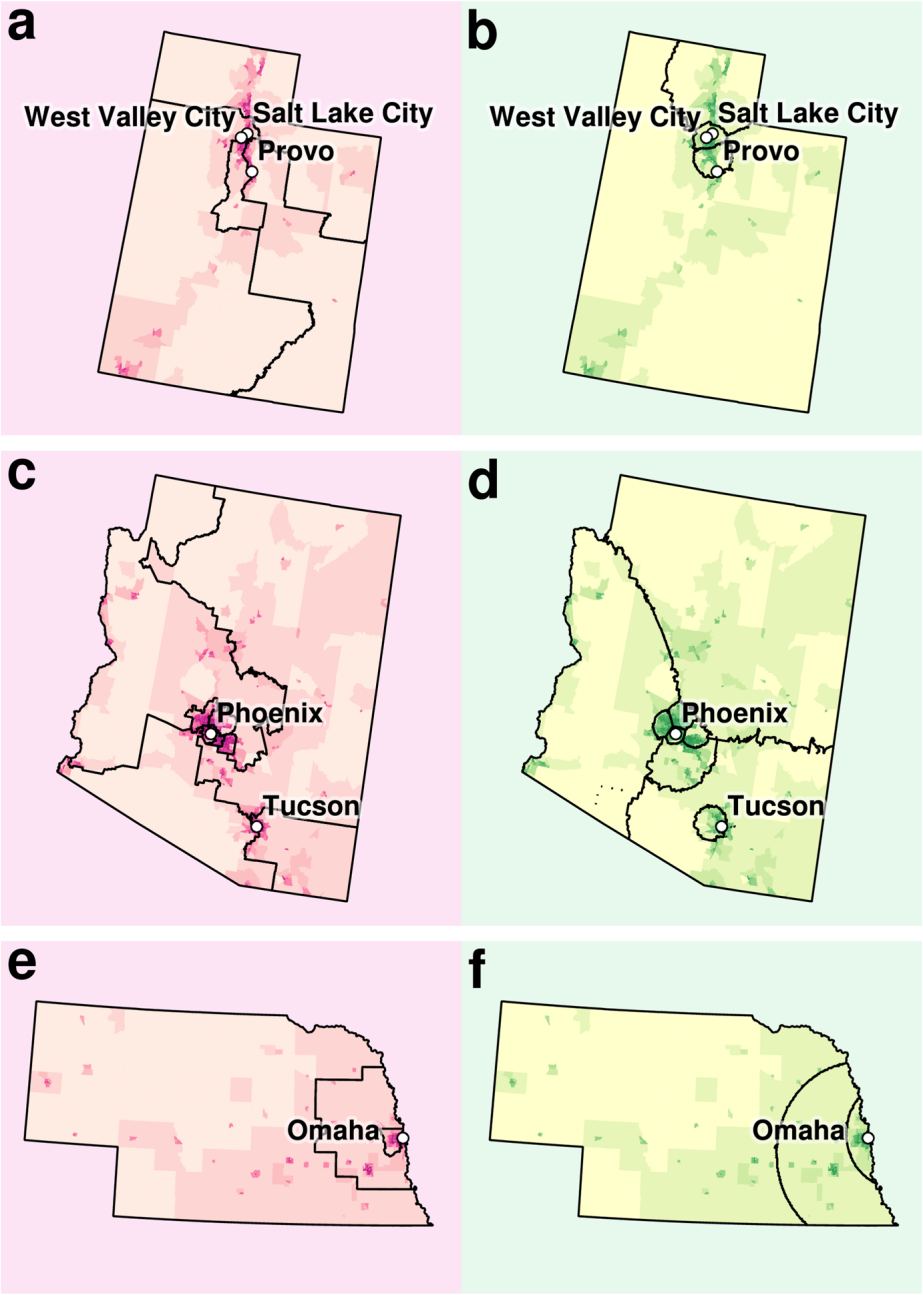
Actual and computed district maps for Utah (**a**, **b**), Arizona (**c**, **d**), and Nebraska (**e**, **f**). Computed solutions are shown in green to the right of the actual congressional districts. Darker areas on the map (census tracts) are more densely populated

Notwithstanding Utah’s “long tradition of requiring that districts be [...] reasonably compact” [10], the densely populated northern conurbation of Provo, Salt Lake City, and West Valley City, is cracked, diluting the urban vote by recruiting parts of the countryside, reaching to the southern border of the state (Fig. 4a). In the computed solution, the urban area of West Valley and Salt Lake City is assigned to a single urban district, as is Provo and its surrounding conurbation (Fig. 4b).

The automated districting of Arizona showcases an emergent property of our algorithm that human-drawn maps have not displayed, namely that districts can be embedded within one another, such as a small densely populated urban district encircled by a large sparsely populated rural district (i.e. shaped like a doughnut). Rather than crack Tucson across three districts (Fig. 4c), the algorithm settled on a doughnut structure (Fig. 4d).

An interesting case of convergence between human and algorithm is the case of Nebraska (Fig. 4e). Our algorithm followed in the footsteps of those who districted Nebraska (Fig. 4f), capturing the same transition from fully urban (east) to fully rural (west). However, the smooth radiating boundaries surrounding the capital, Omaha, are more compact (optimised) in the automated solution.

One question is which solution potential voters prefer. From the 15–17th of November 2017, we collected data from 367 self-identified US citizens (using our website http://redistrict.science) who indicated whether they preferred our algorithmic solution or the actual districting for their state. Participants were asked to select their location using a list of valid US states, then they were shown two images depicting the relevant maps (their own state’s and the one produced by our algorithm) and they had to select which they preferred. The vast majority of respondents preferred the computational solution (90.7% overall; 91.1% when IP address location and state matched) with the pattern holding across states.

## Discussion

In summary, we applied our novel weighted *k*-means algorithm to US Census Bureau data to redistrict the USA’s 435 congressional districts and compared the computed solutions to actual districts. The results confirmed our prediction that larger states would tend to show greater improvement, suggesting that the complexity of the districting task may overwhelm humans’ ability to find optimal solutions. One startling conclusion is that some of what we view as purposeful gerrymandering may reflect human cognitive limitations. At this juncture, this conclusion is more a provocative conjecture than an established finding. Further work is needed to evaluate how human cognitive biases and limitations may contribute to gerrymandering.

In light of our results, we advocate a division of labour between human and machine. Stakeholders should openly debate and justify the districting criteria. Once the criteria are determined by humans, it should be left to the computers to draw the lines given humans’ cognitive limitations and potential partisan bias. We offer one of many potential solutions. The computer code, like ours used in these simulations, should be open source (to allow for replication and scrutiny) and straightforward to provide confidence in its operation.

Political, ethical, scholarly, and legal debate should play a central role in determining the optimisation criteria. For example, instead of choosing the mean pairwise distance between constituents, we could have used travel time to capture the effects of geographical barriers, such as rivers. Even a measure as simple as travel time raises a number of ideological considerations that should be debated, such as the mode of transportation (e.g. public, on foot, or by automobile) to adopt. Other factors could be included in the criteria, such as respecting municipal boundaries, historic communities, the racial composition of districts, partisan affiliation, etc. For our demonstration, we chose perhaps the simplest reasonable criteria, but in application the choice of criteria would ideally involve other factors after lengthy debate involving a number of stakeholders. These debates should elevate democratic discourse by focusing minds on principles and values, as opposed to how to draw maps for partisan advantage.

Although we focused on US districting, similar issues arise in other democracies. For example, the UK is currently reviewing the boundaries for its parliamentary constituencies. Our work suggests that, even though the UK uses politically neutral commissions to guide the redistricting process, the results could disadvantage certain voters due to the cognitive limitations of those drawing the maps.

Our algorithm is only one possible solution to open and automated districting. The algorithm selected could be the one that best performs according to an objective criteria. Different algorithms will provide qualitatively different geometries, which itself could inform selection. For example, the shortest splitline algorithm recursively splits a state into districts restricting itself to north–south and east–west straight lines. The balanced *k*-means algorithm [7] is very similar to our own algorithm. It minimises the standard *k*-means loss function plus an additional weighted term that takes into account the number of members (i.e. people) in each cluster (i.e. district). The range of possible geometries in balanced *k*-means is between those of the shortest splitline algorithm and our weighted *k*-means. Balanced *k*-means will create district boundaries that are lines (at any angle, not just north–south and east–west) to partition the space into a Voronoi diagram. In contrast, our algorithm, which weights distance by cluster, can form districts within districts (see Fig. 4) and borders can be curved (see Fig. 4). No matter the choice of algorithm, clustering is an NP-hard problem such that the optimal solution is not guaranteed unless all possible assignments are considered [19], which is computationally impossible in most cases. In practice, random restart with different initial conditions and other optimisation techniques can provide high-quality solutions.

We believe this automated, yet inclusive and open, approach to redistricting is preferable to the current system in the USA for which the populace’s only remedy is the court system, which has proven ineffective in this arena. The law and case history for gerrymandering in the USA is complex and we will not feign to provide an adequate review here. However, two key points are (a) courts are reactive and proceed slowly relative to the pace of election cycles (i.e. before any action would be taken, disenfranchisement would have already occurred); (b) the Supreme Court of the United States has never struck down a politically gerrymandered district [17]. However, recently, courts have taken a more active role in addressing cases of gerrymandering. After centuries of gerrymandering complaints, for the first time, the Supreme Court has agreed to hear a case concerning whether Wisconsin’s partisan gerrymandering is in breach of the First Amendment and the Voting Rights Act [17]. Likewise, recent verdicts concerning districting in North Carolina and Pennsylvania highlight a growing consensus that politicians should not have a freehand in drawing maps for partisan advantage.

In such legal cases, the concept of voting efficiency, along with comparison to randomly generated maps [9], has prominently featured [25]. The basic concept is that votes for the losing party in a district are “wasted” (related to cracking) as well as votes for the winning party over what is needed to secure victory (related to packing). Formal measures of efficiency can be readily calculated and compared [25]. Although these measures have their place in illustrating disparities, we find it preferable to focus on optimising core principles and values, rather than rarify the status quo and reduce voters to partisan apparatchiks whose preferences and turnout tendencies are treated as fixed across election cycles, which they are not.

In contrast to voter efficiency approaches, an algorithm like ours will naturally lead to cases where groups “self-gerrymander”, such as when like-minded communities form in densely populated areas [8, 21]. However, it is debatable whether these votes are truly wasted. Representatives for these relatively homogeneous communities may have a stronger voice and feel emboldened to advocate for issues that are important to their community, even when these positions may not be popular on the national stage. After all, almost by definition, every important social movement, such as the Civil Rights movement or campaigns for LGBT equality, is not popular at inception. Nevertheless, concepts like voter efficiency could be included in the optimisation criteria for algorithms like ours. When faced with complex issues as to what is fair, the best solution may be the division of labour what we advocate: humans formalise objective criteria through open discourse and the computers search for an optimal solution unburdened by human limitations.
